# Standardized Reporting of Prostate MRI: Comparison of the Prostate Imaging Reporting and Data System (PI-RADS) Version 1 and Version 2

**DOI:** 10.1371/journal.pone.0162879

**Published:** 2016-09-22

**Authors:** Susanne Tewes, Nikolaj Mokov, Dagmar Hartung, Volker Schick, Inga Peters, Peter Schedl, Stefanie Pertschy, Frank Wacker, Götz Voshage, Katja Hueper

**Affiliations:** 1 Institute for Diagnostic and Interventional Radiology, Hannover Medical School, Hannover, Germany; 2 Institute for Diagnostic and Interventional Radiology, Klinikum der Region Hannover, Hannover, Gehrden, Germany; 3 Clinic for Urology, Klinikum der Region Hannover, Hannover, Gehrden, Germany; 4 Department of Urology and Urologic Oncology, Hannover Medical School, Hannover, Germany; Universitair Medisch Centrum Utrecht, NETHERLANDS

## Abstract

**Introduction:**

Objective of our study was to determine the agreement between version 1 (v1) and v2 of the Prostate Imaging Reporting and Data System (PI-RADS) for evaluation of multiparametric prostate MRI (mpMRI) and to compare their diagnostic accuracy, their inter-observer agreement and practicability.

**Material and Methods:**

mpMRI including T2-weighted imaging, diffusion-weighted imaging (DWI) and dynamic contrast-enhanced imaging (DCE) of 54 consecutive patients, who subsequently underwent MRI-guided in-bore biopsy were re-analyzed according to PI-RADS v1 and v2 by two independent readers. Diagnostic accuracy for detection of prostate cancer (PCa) was assessed using ROC-curve analysis. Agreement between PI-RADS versions and observers was calculated and the time needed for scoring was determined.

**Results:**

MRI-guided biopsy revealed PCa in 31 patients. Diagnostic accuracy for detection of PCa was equivalent with both PI-RADS versions for reader 1 with sensitivities and specificities of 84%/91% (AUC = 0.91 95%CI[0.8–1]) for PI-RADS v1 and 100%/74% (AUC = 0.92 95% CI[0.8–1]) for PI-RADS v2. Reader 2 achieved similar diagnostic accuracy with sensitivity and specificity of 74%/91% (AUC = 0.88 95%CI[0.8–1]) for PI-RADS v1 and 81%/91% (AUC = 0.91 95%CI[0.8–1]) for PI-RADS v2. Agreement between scores determined with different PI-RADS versions was good (reader 1: κ = 0.62, reader 2: κ = 0.64). Inter-observer agreement was moderate with PI-RADS v2 (κ = 0.56) and fair with v1 (κ = 0.39). The time required for building the PI-RADS score was significantly lower with PI-RADS v2 compared to v1 (24.7±2.3 s vs. 41.9±2.6 s, p<0.001).

**Conclusion:**

Agreement between PI-RADS versions was high and both versions revealed high diagnostic accuracy for detection of PCa. Due to better inter-observer agreement for malignant lesions and less time demand, the new PI-RADS version could be more practicable for clinical routine.

## Introduction

The use of multiparametric (mp) MRI for the detection and characterization of prostate lesions has evolved over the last 10 years. mpMRI protocols combining information of morphology with high spatial resolution (T2-weighted turbo spin echo imaging = T2 TSE), cell density (diffusion weighted imaging = DWI) and vascularization (dynamic contrast-enhanced imaging = DCE) provide high diagnostic accuracy for the detection of clinically significant prostate cancer (PCa) [1–8]. In addition, MRI is increasingly used for targeted prostate biopsy, which leads to improved detection of significant PCa [9–11].

Consensus has been reached that standardization of imaging and reporting of prostate MRI is important to ensure high diagnostic quality, reproducible MRI results and applicability of prostate MRI for multicenter studies. In 2012 an expert consensus group of the European Society of Urogenital Radiology (ESUR) introduced the version 1 (v1) of the Prostate Imaging Reporting and Data System (PI-RADS) [12]. Since then, PI-RADS v1 has been clinically applied and evaluated in several clinical studies. mpMRI PI-RADS v1 scores showed high diagnostic accuracy for the detection of PCa and high inter-observer agreement [13–16]. However, some limitations of PI-RADS v1 have been recognized in the past years. With this first version, it was not clearly defined how exactly scores should be determined and combined and this gave room for individual interpretation and therefore to variability in the application of PI-RADS v1 [13]. Additionally, various types of perfusion curves that can occur in the prostate led to confusion; there is great heterogeneity in enhancement characteristics of prostate cancers and there is also great heterogeneity in perfusion characteristics of benign prostate lesion [17]. Including perfusion scores in the overall PI-RADS led to higher scores in benign lesions. Therefore, the American College of Radiology (ACR), the ESUR, and the AdMeTech Foundation established a Steering Committee to further develop, update and simplify PI-RADS under consideration of ongoing research in an effort to make PI-RADS more globally acceptable. This resulted in the updated PI-RADS version 2 (v2) [17].

The purpose of this study was to determine the agreement between PI-RADS v1 and v2 for evaluation of multiparametric prostate MRI and to compare their diagnostic accuracy, their inter-observer agreement and practicability.

## Material and Methods

### Patients

Data of 69 consecutive patients who underwent mpMRI of the prostate and subsequently an MRI-guided in-bore biopsy between December 2012 and December 2014 were retrospectively analyzed. 15/69 patients were excluded from the analysis due to an incomplete MRI protocol or distinct artifacts in one or more MRI sequences. Out of 54 patients, 18 patients had at least one negative pre-biopsy and 36 had no pre-biopsy. Mean age (±standard deviation) was 69.6±9.6 years, mean PSA was 8.7±4.9 μg/L and mean prostate volume was 52.1±24.7 ml. The study was approved by the ethics committee of the Hannover Medical School.

### Multiparametric MRI

Multiparametric MRI was acquired according to ESUR guidelines [1, 12] on a 3 Tesla system (Magnetom Skyra, Siemens Healthcare, Erlangen, Germany) using an 18-channel body coil and a spine coil. In order to reduce bowel movement, all patients received an intravenous injection of 20 mg butylscopolamine (Buscopan 20 mg, Boehringer, Ingelheim, Germany) prior to the examination. T2 TSE sequences were acquired in transverse, sagittal and coronal orientation with FOV = 220 x 220 mm^2^, matrix = 300 x 512, TR > 3000 ms, TE > 90 ms. For DWI four b-values = 0, 200, 400, ≥800 s/mm^2^ were used. Other sequence parameters were FOV = 320 x 320 mm^2^, matrix = 120 x 160, TR > 4500 ms and TE > 70 ms. For calculation of ADC maps, a monoexponential model was used and all b-values were included. For DCE a vibe sequence was acquired in transverse plane: FOV = 260 x 260 mm^2^, matrix = 135 x 190, TR = 5 ms, TE = 1.5 ms, temporal resolution = 7.2 s using Gadovist as contrast agent in a weight-adapted standard dose of 0.1 mmol/kg bodyweight with an injection rate of 2.7 ml/s (Table 1).

**Table 1:** Parameters of mpMRI.

	T2 TSE	DWI	DCE (T1 vibe)
TR (ms)	>3000	>4500	5
TE (ms)	>90	>70	1.5
FoV (mm^2^)	220x220	320x320	260x260
matrix	300x512	120x160	135x190
slice thickness (mm)	3mm	4mm	3.6mm
b-values (s/mm^2^)	-	0, 200, 400, ≥800	-

DCE = dynamic contrast enhancement, DWI = diffusion weighted imaging, TSE = turbo spin echo.

### MRI-guided in-bore biopsy

The MRI-guided in-bore biopsy was performed on the same MRI system as diagnostic imaging (Magnetom Skyra, Siemens Healthcare, Erlangen, Germany) using an 18-channel body coil and a spine coil integrated into the scanner. Patients with high clinical probability of PCa and/or equivocal or suspicious MRI findings, as diagnosed by the radiologists who primarily reported the study, underwent biopsy. 65 prostate lesions in 54 patients were successfully biopsied. Patients were placed in prone position. A needle guide connected to the arm of a portable biopsy device (Dyna-TRIM Device, Invivo International PC Best, Netherlands) was inserted rectally after topic local anesthesia (Lidocaine, Instillagel 40 ml, Farco-Pharma, Köln, Germany) was applied. T2-weighted images (HASTE or TSE) were acquired in transverse and sagittal planes. Biopsy was planned using the Dyna-CAD workstation and Dyna-CAD software (Invivo International PC Best, Netherlands) and the determined needle position was adjusted manually. 2 cores were taken per lesion with an MRI-compatible 18-gauge fully automatic biopsy gun. The needle position inside the lesion was verified using T2 HASTE sequences. No additional systematic biopsy was performed.

### PI-RADS analysis

65 prostate lesions in 54 patients were analyzed independently by two readers with five years (reader 1) and 2 years (reader 2) experience in the interpretation of prostate MRI using Visage 7.1 software (Visage Imaging GmbH, Berlin Germany) and a standardized hanging protocol. Readers were blinded to clinical findings and histopathology. Image data of all patients were interpreted twice: once according to PI-RADS v1 [12, 18, 19] and once according to PI-RADS v2 [17]. The time difference for assessment of data in the same patient was at least two weeks. Half of the MRI examinations were first interpreted according to PI-RADS v1, the other half were first interpreted according to PI-RADS v2 in order to avoid bias. In brief, lesions were scored in each sequence (T2 TSE, DWI and DCE) on a scale from 1 = highly likely to be benign to 5 = highly likely to be malignant. For scoring of DCE the presence of focal or asymmetric contrast enhancement as well as the shape of the signal intensity-time curve was evaluated. Signal intensity-time curves were created using Visage 7.1 software by placing a region-of-interest into the suspicious lesion [12]. PI-RADS sum score was calculated as the sum of PI-RADS scores from these sequences. The global PI-RADS score was then determined as follows:

**Table:** 

PI-RADS sum = 3–4	global PI-RADS 1 = highly likely to be benign
PI-RADS sum = 5–6	global PI-RADS 2 = likely to be benign
PI-RADS sum = 7–9	global PI-RADS 3 = indeterminate
PI-RADS sum = 10–12	global PI-RADS 4 = likely to be malignant
PI-RADS sum = 13–15	global PI-RADS 5 = highly likely to be malignant.

PI-RADS v2 analysis was performed according to the recently published guidelines [17]: lesions were categorized into transitional zone (tz) and peripheral zone (pz) lesions. tz lesions were scored on T2 TSE sequences on a scale from PI-RADS 1 to 5. T2 PI-RADS scores of 1–2 and 4–5 accorded with the overall PI-RADS score. Only for intermediate lesions (PI-RADS 3) on T2 TSE images DWI as a second sequence was analyzed. A PI-RADS score of 1–4 on the DWI resulted in an overall PI-RADS score of 3, while a DWI PI-RADS score of 5 results in an overall PI-RADS score of 4. pz lesions were scored primarily based on DWI. Similarly, DWI PI-RADS scores of 1–2 or 4–5 accorded with the overall PI-RADS score. For intermediate lesions (PI-RADS 3) based on DWI, the DCE sequence was visually analyzed and interpreted as positive or negative. DCE was positive when there is enhancement that is focal, earlier or contemporaneous with enhancement of adjacent normal prostatic tissue and corresponds with T2 or DWI lesion [17]. Positive DCE results in an overall PI-RADS of 4, while negative DCE results in an overall PI-RADS of 3 [17]. Additionally, ADC-values were documented. Prostate volume was calculated according to PI-RADS v2 guidelines using the formula for a conventional prolate ellipse (maximum AP diameter x maximum transverse diameter x maximum longitudinal diameter x 0.52) [17].

### Practicability of PI-RADS versions (time need)

In order to evaluate practicability of PI-RADS v2 compared to PI-RADS v1, time was taken for the determination of the PI-RADS scores for each version. Time was measured after all sequences were inspected and lesions were identified and reflects only the time for assigning the PI-RADS score itself.

### Statistical analysis

For statistical analysis, GraphPad Prism software versions 5 (GraphPad Software, Inc., USA) as well as SPSS Statistics version 21 (SPSS, IBM, Chicago, IL, USA) were used. As clinical data and PI-RADS scores were not normally distributed as determined by the Kolmogorov-Smirnov test and PI-RADS scores represent ordinal variables, patients with and without biopsy-proven PCa were compared using the non-parametric Mann-Whitney U test. ADC values were normally distributed and were compared with the unpaired t-test between groups with and without PCa. The time needed for assigning the PI-RADS was not normally distributed and were compared with the non-parametric Wilcoxon test.

Diagnostic performance of mpMRI PI-RADS scores was determined for the dominant lesion in each patient that was biopsied by MRI-guided in-bore biopsy. Results of MRI-guided biopsy were considered as the reference for this study. Receiver operating characteristic (ROC) curve analysis for PI-RADS v1 and v2 was performed separately for each reader as well as for the tz and pz, using histopathological results of MRI-guided in-bore biopsy as the gold standard. Youden-selected thresholds were determined, and sensitivity and specificity of MRI PI-RADS scores at the threshold were recorded. The agreement between PI-RADS versions and the inter-observer agreement for each version was determined using Cohen’s kappa statistics. The agreement was defined excellent (κ>0.81), good (κ = 0.61–0.80), moderate (κ = 0.41–0.60), fair (κ = 0.21–0.40) and poor (κ≤0.20) [20]. Values are given as mean ± standard deviation (SD). P-values <0.05 were considered statistically significant.

## Results

### Comparison of patient data and MRI parameters with and without PCa

**Fig 1:**
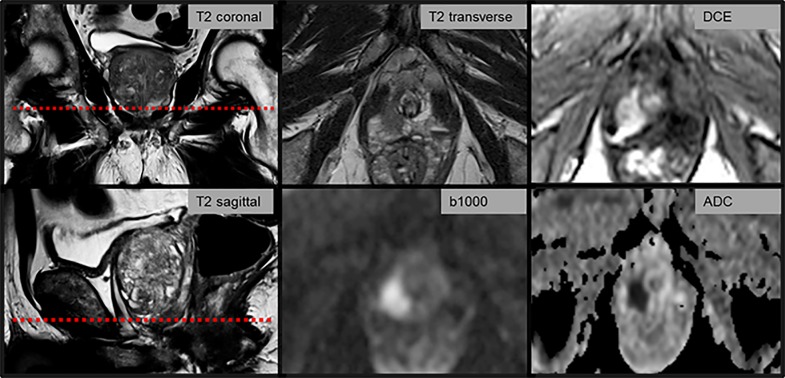
Example of PI-RADS scoring with version 1 and 2 in a patient with prostate cancer. mpMRI of an 82-year-old man with elevated PSA of 8.2 μg/l. The dominant lesion is located in the apex of the prostate in the pz and measures 12 x 18 x 17 mm. According to PI-RADS v2, DWI is the leading sequence. As the lesion has high signal intensity on b1000 with corresponding strong ADC reduction of 0.5 10^−3^ mm^2^/s as well as a diameter >15 mm the lesion was scored PI-RADS 5, highly likely to be malignant. No other sequence is required for scoring according to PI-RADS v2. With PI-RADS v1 a score for each sequence needs to be determined. For this patient the following scores were assigned: T2 TSE PI-RADS 5, DWI PI-RADS 5, DCE PI-RADS 5. This results in in a PI-RADS sum of 15 and a global PI-RADS 5. In this patient the time need for PI-RADS scoring with v2 after inspection of all images was 9 seconds, while it was 59 seconds with v1. MRI-guided in-bore biopsy revealed Gleason 3+4 = 7 tumor.

MRI-guided in-bore biopsy revealed PCa in 31/54 patients (57%), 3 patients with Gleason 9, 1 patient with Gleason 8, 3 patients with Gleason 7b, 4 patients with Gleason 7a, 20 patients with Gleason 6. According to D’Amico criteria, 8 out of 20 patients with Gleason 6 had significant prostate cancer. In 40 patients the dominant lesion was located in the pz; 26/40 of these lesions were positive for PCa. In 14 patients the dominant lesion was located in the tz; 5/14 of these were positive for PCa. No significant difference in PSA level was observed in patients with and without PCa (9.3±5.9 vs. 7.8±3.3 μg/l, p = 0.3). Prostate volume was significantly lower in the group with PCa (46.9±25.8 vs. 59.2±21.6 ml, p<0.05). PI-RADS scores were higher in patients with histologically proven PCa with both PI-RADS versions (PI-RADS v1: 4.3±0.8 vs. 2.4±1.0, p<0.001; PI-RADS v2: 4.2±0.7 vs. 2.3±1.0, p<0.001). ADC values were significantly lower in tumor-positive lesion (0.8±0.2 vs. 1.2±0.2 10^−3^ mm^2^/s, p<0.001, Table 2). An example of PI-RADS scoring in a patient with biopsy proven PCa (Gleason 3+4 = 7a) is given in Fig 1. An overview of PI-RADS scores assigned according to PI-RADS version 1 and version 2 is given in Tables 3 and 4.

**Table 2:** Comparison of patient data and MRI parameters in patients with and without prostate cancer.

	parameter	patients with histologically proven PCa n = 31	patients without histologically proven PCa n = 23	p-value
patient data	age (years)	72.6±8.8	65.4±9.2	<0.01
	PSA (μg/L)	9.3±5.9	7.8±3.3	0.3
MRI parameters	Prostate volume (ml)	46.9±25.8	59.2±21.6	<0.05
	PI-RADS v1	4.3±0.8	2.4±1.0	<0.001
	PI-RADS v2	4.2±0.7	2.3±1.0	<0.001
	ADC value(10^−3^ mm^2^/s)	0.8±0.2	1.2±0.2	<0.001

Values are given as mean ± standard deviation. ADC = apparent diffusion coefficient, DCE = dynamic contrast-enhanced imaging, DWI = diffusion weighted imaging, MRI = magnetic resonance imaging, PCa = prostate carcinoma, PI-RADS = Prostate Imaging Reporting and Data System, PSA = prostate specific antigen.

**Table 3:** PI-RADS scores assigned according to v1.

		Reader 2					all
		PI-RADS 1	PI-RADS 2	PI-RADS 3	PI-RADS 4	PI-RADS 5	
Reader 1	PI-RADS 1	2	1	0	0	0	3
	PI-RADS 2	3	6	2	0	0	11
	PI-RADS 3	1	3	6	2	0	12
	PI-RADS 4	0	0	4	7	1	12
	PI-RADS 5	0	0	1	8	7	16
all		6	10	13	17	8	54

**Table 4:** PI-RADS scores assigned according to v2.

		Reader 2					all
		PI-RADS 1	PI-RADS 2	PI-RADS 3	PI-RADS 4	PI-RADS 5	
Reader 1	PI-RADS 1	1	2	0	0	0	3
	PI-RADS 2	2	9	3	0	0	14
	PI-RADS 3	0	3	3	1	0	7
	PI-RADS 4	0	1	3	15	0	19
	PI-RADS 5	0	0	0	3	8	11
all		3	15	9	19	8	54

### Diagnostic accuracy of mpMRI PI-RADS scores version 1 and version 2

**Fig 2:**
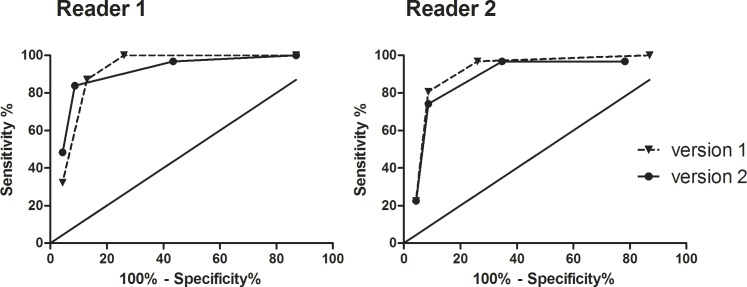
Diagnostic accuracy of PI-RADS scoring with the two versions for experienced and unexperienced readers. ROC-curves show diagnostic accuracy of PI-RADS scoring for an experienced (reader 1) and less experienced reader (reader 2) with PI-RADS version 1 (continuous line) and version 2 (dashed line). Points and triangles correspond to PI-RADS thresholds. Sensitivies and specificities as well as Youden-selected thresholds are given in Table 2.

Sensitivity and specificity for the detection of PCa were 100% and 74%, respectively, at the Youden-selected cut-off PI-RADS ≥3 with v2 and 84% and 91%, respectively, at a Youden-selected cut-off PI-RADS ≥4 with v1 for the experienced reader. Youden-selected thresholds were similar for the less experienced reader, who achieved nearly equivalent sensitivities and specificities with PI-RADS v2 (Table 5, Fig 2).

**Table 5:** Diagnostic accuracy of mpMRI PI-RADS scores version 1 and version 2.

Diagnostic parameter	reader	AUC[95% CI]	Youden selected threshold	sensitivity at threshold	specificity at threshold	Youden index at threshold
PI-RADS v1	1	0.91[0.8;1]	≥4	84%	91%	75%
PI-RADS v1	2	0.88[0.8;1]	≥4	74%	91%	65%
PI-RADS v2	1	0.92 [0.8;1]	≥3	100%	74%	74%
PI-RADSv2	2	0.91 [0.8;1]	≥3	81%	91%	72%

AUC = area under curve, CI = confidence interval, DCE = dynamic contrast-enhanced imaging, PI-RADS = Prostate Imaging Reporting and Data System, DWI = diffusion weighted imaging. Reader 1 represents the experienced reader with 5 years experience in the interpretation of prostate MRI and reader 2 represents the less experienced reader with 2 years experience.

**Fig 3:**
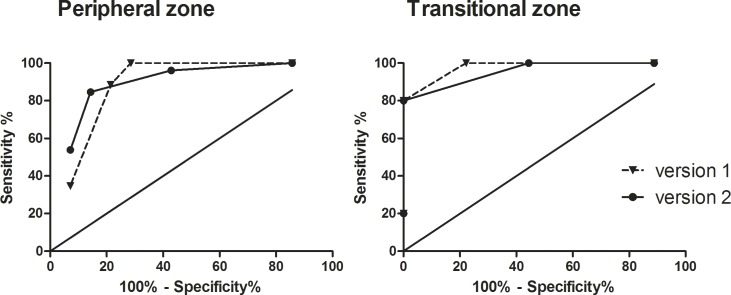
Diagnostic accuracy of PI-RADS scoring with the two versions for peripheral and transitional zone lesions. ROC-curves depict diagnostic accuracy of PI-RADS scoring for peripheral and transitional zone lesions with PI-RADS version 1 (continuous line) and version 2 (dashed line). Points and triangles correspond to PI-RADS thresholds. Sensitivies and specificities as well as Youden-selected thresholds are given in Table 3.

In addition, diagnostic accuracy for pz and tz lesions was analyzed separately. With PI-RADS v2 scores best diagnostic accuracy in the pz was reached with a cut-off ≥3 at a Youden-Index of 71% (sensitivity 100%, specificity 71%), while in the tz a cut-off ≥4 revealed highest diagnostic accuracy (sensitivity 80%, specificity 100%, Youden-Index 80%). With PI-RADS v1 Youden-selected cut-off was ≥4 for both pz and tz lesions (Table 6, Fig 3).

**Table 6:** Diagnostic accuracy of mpMRI PI-RADS scores version 1 and version 2 for peripheral and transition zone lesions.

	Diagnostic parameter	zone	AUC[95% CI]	Youden selected threshold	sensitivity at threshold	specificity at threshold	Youden index at threshold
reader 1	PI-RADS v1	pz	0.89[0.8;1]	≥4	85%	86%	71%
	PI-RADS v1	tz	0.96[0.8;1.1]	≥4	80%	100%	80%
	PI-RADS v2	pz	0.88 [0.8;1]	≥3	100%	71%	71%
	PI-RADSv2	tz	0.97 [0.9;1.1]	≥4	80%	100%	80%
reader 2	PI-RADS v1	pz	0.84[0.7;1.0]	≥3	96%	64%	60%
	PI-RADS v1	tz	0.97[0.9;1.1]	≥4	80	100	80%
	PI-RADS v2	pz	0.88[0.8;1.0]	≥3	96%	79%	75%
	PI-RADSv2	tz	0.97[0.9;1.1]	≥4	80%	100%	80%

AUC = area under curve, CI = confidence interval, DCE = dynamic contrast-enhanced imaging, NPV = negative predictive value, PI-RADS = Prostate Imaging Reporting and Data System, DWI = diffusion weighted imaging, pz = peripheral zone, tz = transition zone.

### Practicability of PI-RADS scoring (time need)

When comparing the time needed to determine the PI-RADS score after having evaluated the entire examination, the experienced reader needed 24.7±2.3 seconds for scoring according to PI-RADS v2 and 41.9±2.6 seconds for PI-RADS v1 (p<0.001). For intermediate lesions (PI-RADS 3), which require analysis of two sequences for PI-RADS v2, no significant difference in the time need was found with 37.1±6.2 seconds for v2 and 48.2±5.1 seconds for v1 (p = 0.2). For PI-RADS 4/5 lesions (19.5±2.1 vs. 38.8±3.1 seconds, p<0.001) as well as for PI-RADS 1/2 lesions (22.5±3.5 vs. 42.1±6.4 seconds, p<0.05) the time need was significantly shorter for PI-RADS v2 compared to v1.

### Agreement between PI-RADS versions and between observers

The agreement between PI-RADS v1 and v2 scores was good for both readers for all lesions (reader 1: κ = 0.62, reader 2: κ = 0.64), moderate when considering only malignant lesion (reader 1: κ = 0.54, reader 2: κ = 0.53) and moderate when for benign lesions (reader 1: κ = 0.54, reader 2: κ = 0.60).

The agreement between the experienced and less experienced reader (inter-observer agreement) with PI-RADS v1 was fair (κ = 0.39) for all lesions, poor for biopsy proven malignant lesions (κ = 0.14) and moderate for benign lesions (κ = 0.50). With PI-RADS v2 the agreement was moderate for all lesions (κ = 0.56) and for malignant lesions (κ = 0.56) and fair for benign lesions (κ = 0.26).

## Discussion

We showed that the updated PI-RADS v2 for evaluation of mpMRI of the prostate had high diagnostic accuracy for detection of PCa with equivalent sensitivities and specificities compared to PI-RADS v1. The agreement between PI-RADS v1 and v2 scores was good. The inter-observer agreement for malignant lesions was better with PI-RADS v2 than with PI-RADS v1 and the time needed for PI-RADS scoring was significantly shorter for PI-RADS v2 indicating a better practicability.

Rising acceptance of mpMRI by urologists depends on high diagnostic accuracy for detection of significant PCa and reproducible interpretation. Therefore, standardized analysis and reporting of prostate MRI with comprehensible and clearly defined criteria are required. The initial version of the PI-RADS scoring (version 1) revealed high diagnostic accuracy in several studies [13] and good inter-observer agreement [15]. In the present study, comparing the updated PI-RADS scoring (version 2) with the initial version 1, we found equivalent diagnostic accuracy and a good agreement between the PI-RADS versions. The updated PI-RADS v2 provides a more detailed description on the assessment of prostate MRI with clearly defined criteria for PI-RADS scoring and representative images for each PI-RADS score and each sequence separately for pz and tz lesions [17]. As it was done with PI-RADS v1, the complete mpMRI examination (T2, DWI and DCE) has to be acquired and inspected completely. Major renewals in PI-RADS v2 are that the relevance of MRI-sequences is weighted depending on the localization of the lesion in the pz or tz and that scoring in one sequence is sufficient for most lesions. Only for indeterminate lesions (PI-RADS 3) scoring in a second sequence is required. In PI-RADS v2 the most important sequence for diagnosis of significant PCa is DWI, being the leading sequence for the pz and the second sequence for the tz. This development is based on previous research showing that DWI provides highest accuracy for PCa detection for pz and tz lesions, if only one sequence is considered [21–23]. For tz tumors the combination of T2 and DWI, as used in PI-RADS v2, has been reported to have highest diagnostic accuracy [21], while adding DCE could not improve tumor detection in treatment-naïve prostates [24]. In contrast, in the pz diagnostic performance of DCE is better [21, 24], so that it is reasonable to use DCE as a second sequence in PI-RADS v2, if DWI reveals indeterminate results (PI-RADS 3). Besides the opportunity to consider different tissue characteristics of pz and tz with PI-RADS v2, scoring of one or a maximum of two MRI sequences is more straightforward and efficient. Importantly, there are several reasons why it is still recommended for prostate MRI to acquire and interpret morphological T2-weighed images and at least two functional sequences. First, tumor localization and characteristics are unknown before the examination, so that it is not clear which sequences are necessary for PI-RADS v2 scoring. Second, if image quality due to artifacts in one sequence is insufficient (e.g. distortion in DWI often occurs with hip implants), the other two sequences can still be used for PI-RADS scoring, as the new version suggests a score if one sequence is missing [17]. Third, in pre-treated prostates following radiation therapy, focal therapy or endourethral treatment detection of PCa is often more challenging and treatment related signal changes have to be considered. In this situation other sequences than suggested in the PI-RADS v2 might be helpful. Therefore, in our study, PI-RADS scores were assigned after the entire examination was closely evaluated and artifacts were excluded.

Sensitivities and specificities of both PI-RADS versions in our study were comparable to those reported previously for PI-RADS v1 in studies that accurately used PI-RADS scoring and had pooled sensitivity of 82% (95%CI; 0.7–0.9) and specificity of 82% (95% CI; 0.7–0.9) [13–16, 25–28]. In a recent study, 95% of PCa foci ≥0.5 ml were correctly identified with PI-RADSv2 using whole-mount pathology as reference standard [29]. Youden-selected PI-RADS thresholds in our study varied between PI-RADS ≥3 and ≥4 depending on PI-RADS version and tumor localization. The calculated threshold for best diagnostic accuracy of all lesions with version 2 was ≥3, while it was ≥4 with version 1. However, the number of patients might be too low to draw any further conclusions from this discrepancy between PI-RADS versions. In addition, recommendations for patient management based on mpMRI PI-RADS scores have not been proposed so far and are explicitly not included in the PI-RADS v2 document [17]. Development of reliable recommendations based on PI-RADS scores needs further research with large prospective multicenter trials and long-term follow-up of patients.

The inter-observer agreement between experienced and less experienced readers in our study was moderate for PI-RADS v2 (κ = 0.56) and fair for PI-RADS v1 (κ = 0.39) when considering all lesions. A recent study, evaluating PI-RADS v2, also found moderate interreader agreement for five interpreters when considering all lesions [30]. Rosenkrantz et al. reported that inter-observer agreement (using PI-RADS v1) between experienced readers was better (concordance correlation coefficient 0.61–0.68) than between experienced and inexperienced readers (concordance correlation coefficient 0.34–0.48) for all lesions [31], indicating that reader agreement is dependent on experience with prostate MRI. Schimmoeller et al. showed a good inter-reader agreement for tumor-positive lesions and a moderate to good interreader agreement for benign lesions with PI-RADS v1 [15]. An explanation for the fact that inter-observer agreement is better for tumor-positive lesions might be that various types of benign changes of prostate tissue occur, which are often difficult to differentiate from tumor lesions. In our study, when considering only biopsy-proven PCa lesions, inter-observer agreement with PI-RADS v2 (κ = 0.56) was better than with PI-RADS v1 (κ = 0.14). This may be explained by a more detailed and clear definition of PI-RADS scores with v2. For example PI-RADS 4 and 5 lesions with v2 are clearly differentiated by the lesion size (diameter ≥15 mm results in PI-RADS 5), the presence of definite extraprostatic extension or invasive behavior.

Another important finding of our study is that the time needed for assigning the PI-RADS score itself was significantly shorter with v2 compared to v1, which is due to the fact that for most prostate lesions (i.e. PI-RADS 1,2, 4 and 5 lesions) scoring of only one sequence for v2 instead of three sequences for v1 is necessary. Accordingly, for PI-RADS 3 lesions, requiring two sequences for PI-RADS v2 scoring, the time difference was less pronounced and statistical significance was not reached. Furthermore, producing a signal intensity-time curve is not required for DCE analysis with version 2, which saves additional time. Furthermore, the newly introduced sector map of the prostate with 39 regions is more intuitive as the regions are named according to their location and are not numbered [17]. Therefore, our study suggests that PI-RADS v2 might indeed be more practicable and easier to implement into clinical routine. Note that the time needed to evaluate the entire prostate MRI and to identify the lesions was not included in the time needed for assigning the PI-RADS score.

Limitations of our study are that MRI-guided in-bore biopsy was used as a reference standard, no additional systematic biopsy was performed and no long-term follow-up was available. Therefore, false negative biopsy results cannot be excluded, which has to be considered when interpreting diagnostic accuracy results. However, the primary objective of our work was to directly compare the two PI-RADS versions in terms of inter- and intra-observer variability, practicability and their diagnostic performance.

In conclusion, standardized evaluation of prostate MRI according to PI-RADS v2 showed high diagnostic accuracy for the detection of PCa, equivalent to PI-RADS v1. The agreement between the two PI-RADS versions was good. Of note, inter-observer agreement for tumor-positive lesions was better with PI-RADS v2 and shorter time was needed for scoring according to PI-RADS v2 indicating better practicability for clinical practice.

## Supporting Information

S1 Table Patient details. Pat. no. = patient number, v1 = PI-RADS version 1, v2 = PI-RADS version 2, r1 = reader 1, r2 = reader 2, pca = prostate carcinoma, PSA = prostate specific antigen, pz = peripheral zone, tz = transitional zone, ADC = apparent diffusion coefficient.
(DOCX)
