# Supplementary material for: Standardized Reporting of Prostate MRI: Comparison of the Prostate Imaging Reporting and Data System (PI-RADS) Version 1 and Version 2
Source: PLoS One. 2016 Sep 22;11(9):e0162879. doi: 10.1371/journal.pone.0162879 (PMC5033350; doi:10.1371/journal.pone.0162879)
Supplement: S1 Table — Pat. no. = patient number, v1 = PI-RADS version 1, v2 = PI-RADS version 2, r1 = reader 1, r2 = reader 2, pca = prostate carcinoma, PSA = prostate specific antigen, pz = peripheral zone, tz = transitional zone, ADC = apparent diffusion coefficient. (DOCX) [file pone.0162879.s001.docx]

**S1 Table.** Patient details

| Pat. no. | v1 r1 | v1 r2 | v2 r1 | v2 r2 | pca no=0 yes=1 | PSA ng/ml | Gleason-sum | pz=1 tz=2 | postate volume | length x width x height x 0.52 (ml) | ADC 10^-3^ mm^2^/s | time v1 (sec) | time v2 (sec) |
| --- | --- | --- | --- | --- | --- | --- | --- | --- | --- | --- | --- | --- | --- |
| 1 | 5 | 5 | 4 | 4 | 1 | 14 | 3+3=6 | 1 | 58x40x42 | 51 | 0.725 | . | . |
| 2 | 5 | 4 | 4 | 4 | 1 | 1.3 | 3+3=6 | 1 | 46x35x43 | 36 | 0.912 | . |  |
| 3 | 1 | 2 | 1 | 2 | 0 | 9.7 |  | 1 | 57x49x66 | 96 | 1.299 | 109 | 20 |
| 4 | 4 | 5 | 5 | 5 | 1 | 5.3 | 3+3=6 | 2 | 57x38x38 | 43 | 0.866 | 57 | 20 |
| 5 | 2 | 3 | 3 | 3 | 1 | 5.6 | 3+3=6 | 1 | 51x28x34 | 25 | 1.069 | 68 | 66 |
| 6 | 4 | 4 | 4 | 4 | 1 | 4.0 | 3+3=6 | 1 | 42x29x38 | 24 | 0.864 | 86 | 65 |
| 7 | 3 | 4 | 4 | 4 | 1 | 4.8 | 3+4=7 | 1 | 50x39x37 | 38 | 0.833 | 74 | 64 |
| 8 | 4 | 4 | 4 | 4 | 1 | 13 | 3+3=6 | 1 | 56x30x42 | 37 | 0.794 | 51 | 26 |
| 9 | 5 | 4 | 5 | 4 | 1 | 17 | 3+3=6 | 1 | 47x30x35 | 26 | 1.034 | 42 | 33 |
| 10 | 2 | 2 | 2 | 2 | 0 | 14 |  | 1 | 54x41x42 | 48 | 1.620 | 51 | 29 |
| 11 | 5 | 4 | 5 | 5 | 1 | 9.0 | 4+5=9 | 1 | 54x42x55 | 65 | 0.697 | 49 | 19 |
| 12 | 2 | 2 | 2 | 2 | 0 | 4.7 |  | 2 | 51x36x41 | 39 | 1.645 | 54 | 26 |
| 13 | 5 | 4 | 5 | 4 | 1 | 20.0 | 3+3=6 | 1 | 56x37x36 | 39 | 0.879 | 32 | 19 |
| 14 | 2 | 1 | 1 | 1 | 0 | 11.0 |  | 1 | 46x34x46 | 37 | 1.188 | 45 | 14 |
| 15 | 3 | 3 | 3 | 3 | 0 | 10.0 |  | 2 | 63x54x52 | 92 | 1.159 | 79 | 60 |
| 16 | 1 | 1 | 2 | 1 | 0 | 10.0 |  | 1 | 63x34x44 | 49 | 1.281 | 28 | 9 |
| 17 | 3 | 3 | 3 | 2 | 0 | 6.9 |  | 2 | 50x34x51 | 45 | 0.987 | 60 | 43 |
| 18 | 3 | 2 | 4 | 2 | 0 | 4.0 |  | 1 | 43x29x45 | 29 | 1.326 | 49 | 62 |
| 19 | 5 | 5 | 5 | 5 | 0 | 10 |  | 1 | 60x42x54 | 71 | 0.897 | 68 | 24 |
| 20 | 2 | 2 | 2 | 3 | 0 | . |  | 2 | 56x46x67 | 90 | 0.941 | 44 | 57 |
| 21 | 4 | 4 | 4 | 4 | 1 | 6.7 | 3+3=6 | 2 | 60x36x55 | 62 | 0.779 | 45 | 11 |
| 22 | 3 | 2 | 2 | 2 | 0 | 4.0 |  | 2 | 59x47x47 | 68 | 0.905 | 38 | 14 |
| 23 | 4 | 4 | 4 | 4 | 1 | 5.0 | 3+4=7 | 1 | 41x20x24 | 10 | 0.656 | 29 | 13 |
| 24 | 3 | 2 | 2 | 2 | 0 | 5.7 |  | 1 | 56x37x43 | 46 | 0.855 | 37 | 26 |
| 25 | 4 | 3 | 4 | 4 | 1 | 6 | 3+3=6 | 1 | 49x39x32 | 32 | 1.001 | 31 | 23 |
| 26 | 4 | 4 | 4 | 4 | 1 | 9.1 | 3+3=6 | 1 | 44x29x40 | 27 | 1.154 | 41 | 15 |
| 27 | 5 | 5 | 5 | 4 | 1 | 8.2 | 3+4=7 | 1 | 57x43x58 | 74 | 0.513 | 59 | 9 |
| 28 | 3 | 1 | 3 | 2 | 1 | 11.8 | 3+3=6 | 1 | 49x31x24 | 19 | 1.088 | 47 | 13 |
| 29 | 5 | 4 | 4 | 4 | 1 | 7.4 | 3+3=6 | 1 | 34x48x48 | 41 | 0.823 | 33 | 15 |
| 30 | 3 | 3 | 2 | 3 | 0 | 10 |  | 2 | 64x58x53 | 102 | 0.993 | 28 | 14 |
| 31 | 5 | 3 | 4 | 3 | 1 | 20.8 | 3+3=6 | 1 | 62x52x63 | 106 | 0.842 | 47 | 15 |
| 32 | 2 | 2 | 2 | 2 | 0 | 2.0 |  | 2 | 65x42x54 | 77 | 1.281 | 49 | 27 |
| 33 | 3 | 3 | 2 | 3 | 0 | 4.8 |  | 1 | 58x35x41 | 43 | 0.916 | 60 | 19 |
| 34 | 4 | 4 | 4 | 4 | 0 | 10.1 |  | 1 | 53x51x50 | 70 | 1.031 | 24 | 17 |
| 35 | 3 | 4 | 3 | 4 | 1 | 11.7 | 3+3=6 | 2 | 56x36x44 | 46 | 0.606 | 32 | 61 |
| 36 | 5 | 5 | 5 | 5 | 1 | 7.3 | 3+3=6 | 1 | 47x30x42 | 31 | 0.681 | 36 | 13 |
| 37 | 3 | 3 | 3 | 3 | 1 | 7.0 | 4+3=7 | 1 | 52x60x64 | 104 | 0.686 | 25 | 15 |
| 38 | 2 | 1 | 2 | 1 | 0 | 5.8 |  | 1 | 46x33x43 | 34 | 1.009 | 24 | 29 |
| 39 | 2 | 1 | 2 | 2 | 0 | 9.3 |  | 2 | 53x45x55 | 68 | 1.273 | 28 | 19 |
| 40 | 2 | 2 | 2 | 2 | 0 | 5.8 |  | 1 | 48x39x49 | 48 | 1.158 | 40 | 25 |
| 41 | 4 | 3 | 4 | 3 | 1 | 8.0 | 3+3=6 | 2 | 46x35x39 | 33 | 0.846 | 44 | 18 |
| 42 | 4 | 4 | 4 | 4 | 1 | 8.0 | 3+4=7 | 1 | 60x37x46 | 53 | 0.967 | 24 | 20 |
| 43 | 4 | 3 | 4 | 4 | 1 | 4.9 | 4+4=8 | 1 | 42x29x35 | 22 | 0.600 | 29 | 18 |
| 44 | 5 | 4 | 5 | 5 | 1 | 8.0 | 4+5=9 | 1 | 40x56x53 | 62 | 0.691 | 23 | 16 |
| 45 | 1 | 1 | 1 | 2 | 0 | 5.4 |  | 2 | 51x42x43 | 48 | 1.333 | 23 | 8 |
| 46 | 5 | 5 | 5 | 5 | 1 | 5.5 | 4+3=7 | 1 | 42x33x50 | 36 | 0.650 | 26 | 17 |
| 47 | 5 | 4 | 4 | 4 | 1 | 4.6 | 3+3=6 | 1 | 57x41x47 | 57 | 0.687 | 31 | 13 |
| 48 | 3 | 3 | 3 | 2 | 0 | 6.0 |  | 1 | 49x36x45 | 41 | 0.882 | 29 | 25 |
| 49 | 5 | 5 | 4 | 4 | 1 | 6.2 | 3+3=6 | 2 | 51x36x47 | 39 | 0.706 | 22 | 17 |
| 50 | 5 | 5 | 5 | 5 | 1 | 28.0 | 3+3=6 | 1 | 46x30x41 | 29 | 0.707 | 31 | 15 |
| 51 | 4 | 3 | 4 | 3 | 1 | 12.0 | 5+4=9 | 1 | 56x43x55 | 69 | 0.722 | 21 | 21 |
| 52 | 2 | 2 | 2 | 2 | 0 | 9.0 |  | 1 | 49x39x45 | 45 | 1.450 | 24 | 14 |
| 53 | 2 | 3 | 2 | 2 | 0 | 14.0 |  | 1 | 60x47x51 | 75 | 0.953 | 28 | 16 |
| 54 | 5 | 4 | 5 | 5 | 1 | . | 4+3=7 | 1 | 65x51x68 | 117 | 0.886 | 27 | 16 |

Pat. no. = patient number, v1 = PI-RADS version 1, v2 = PI-RADS version 2, r1 = reader 1, r2 = reader 2, pca = prostate carcinoma, PSA = prostate specific antigen, pz = peripheral zone, tz = transitional zone, ADC = apparent diffusion coefficient.
